# A Hybrid CNN Framework DLI-Net for Acne Detection with XAI

**DOI:** 10.3390/jimaging11040115

**Published:** 2025-04-10

**Authors:** Shaila Sharmin, Fahmid Al Farid, Md. Jihad, Shakila Rahman, Jia Uddin, Rayhan Kabir Rafi, Radia Hossan, Hezerul Abdul Karim

**Affiliations:** 1 Department of Computer Science, American International University-Bangladesh, Dhaka 1229, Bangladesh; 2 Centre for Image and Vision Computing (CIVC), COE for Artificial Intelligence, Faculty of Artificial Intelligence and Engineering (FAIE), Multimedia University, Cyberjaya 63100, Selangor, Malaysia; fahmid.farid@gmail.com; 3 Department of Computer Science & Engineering, East West University, Dhaka 1212, Bangladesh; mdjihad.ewu@gmail.com; 4 Artificial Intelligence and Big Data Department, Woosong University, Daejeon 34606, Republic of Korea; jia.uddin@wsu.ac.kr; 5 Department of Electrical and Electronic Engineering, American International University-Bangladesh, Dhaka 1229, Bangladesh; 6 Department of Information Technology, University of Information Technology and Sciences, Dhaka 1212, Bangladesh

**Keywords:** image classification, deep learning, acne detection, image segmentation, explainable AI, DLI-Net

## Abstract

Acne is a prevalent skin condition that can significantly impact individuals’ psychological and physiological well-being. Detecting acne lesions is crucial for improving dermatological care and providing timely treatment. Numerous studies have explored the application of deep learning models to enhance the accuracy and speed of acne diagnoses. This study introduces a novel hybrid model that combines DeepLabV3 for precise image segmentation with InceptionV3 for classification, offering an enhanced solution for acne detection. The DeepLabV3 model isolates acne lesions and generates accurate segmentation masks, while InceptionV3 efficiently classifies the different types of acne, improving the overall diagnostic accuracy. The model was trained using a custom dataset and evaluated using advanced optimization techniques. The hybrid model achieved exceptional performances with a validation accuracy of 97%, a test accuracy of 97%, an F1 score of 0.97, a precision of 0.97, and a recall of 0.97, surpassing many of the existing baseline models. To enhance its interpretability further, Grad-CAM (Gradient-Weighted Class Activation Mapping) is utilized to visualize the regions of the image that the model focuses on during predictions, providing transparent insights into the decision-making process. This study underscores the transformative potential of AI in dermatology, offering a robust solution for acne detection and classification, which can significantly improve clinical decision making and patient outcomes.

## 1. Introduction

Acne vulgaris is a common skin disorder among adolescents and young adults, and an exact diagnosis and classification are imperative for advising the proper treatment. Grading acne often depends upon a clinician’s acumen; it is subjective and prone to variability. One’s clinical acumen depends on one’s experience, and this dependency usually raises the need for objective and standardized methods.

Facial skin disorders, such as acne, are among the most studied themes because if they are left untreated, they can lead to scars and therefore must be diagnosed as early as possible [1]. Conventionally, acne detection is performed by identifying color or texture features and finding classifiers. The obstacles that arise due to the feature diversity and threshold settings are now being overcome using deep learning. The segmentation improves when including labeled and synthetic data with semi-supervised learning and generative models. Facial skin problems, especially acne, are of great concern for millions worldwide, since 9.4% of the population faces this issue and experiences its physically and mentally challenging processes [2]. However, Camera-based facial image analyses, now supported by modern computing and high-resolution cameras, promise applications beyond security to include medically relevant diagnostics such as skin lesion assessments for conditions like acne [3]. While many challenges arise in image processing because of image artifacts and variable conditions, more recent research proposed frameworks that combine lesion and healthy skin contexts to improve the detection accuracy for clinical support in early diagnosis and treatment. Acne vulgaris is the most frequent skin disorder, originating from an intricate interplay between androgens, keratinocyte proliferation, and sebum production with bacterial infiltration and inflammation [4]. Acne seriously affect patients’ mental health, varying by different measures. Recent and profound learning techniques have used transfer learning models like DenseNet121. Meanwhile, explainable AI tools like LIME have used automated acne classification and visualization for diagnostic accuracy. Acne vulgaris is a condition that affects many populations around the world, bringing economic hardship and psychological stress; therefore, its diagnosis urgently needs updating, especially regarding the shortage of dermatologists [5]. Recent and profound learning studies have improved the accuracy of detection and grading for acne with new models such as CenterNet and EfficientNet-b4, enabled by smartphone-based image capture and explainable AI, toward a better diagnostic performance, increased speed, and accessibility. Skin lesions, including acne vulgaris, represent some of the most challenging diagnoses; considering the level of complexity, they are overwhelmingly time consuming, not considering the traditional methods, which calls for an urgent need for efficiency in automation [6]. Recent advancement within deep learning and IoT technologies enabled a state-of-the-art dermatological system, “AC-Skin”, which streamlines the data acquisition, improves the diagnostic performance, and bridges research gaps in the automated detection of facial acne and measurement of its severity. Acne vulgaris is an inflammatory skin disease that results from the obstruction of the hair follicles and sebaceous glands and is usually present as several forms of lesions, including comedones, papules, pustules, nodules, and cysts [7]. An artificial intelligence model was built for the detection and classification of this diagnostic burden at a precision of 12 subclasses of acne lesions based on the YOLOv8 architecture. This could help dermatologists develop the best treatment strategy for their patients. Acne vulgaris is the most common skin disorder, founded on inflammation of the hair follicles, especially in parts of the body with high numbers of oil glands. Quality of life can be seriously affected by both scarring and its psychological effects [8]. Deep-learning approaches to acne detection using hyperspectral imaging are a new development in this field. It is worth noting that these approaches enable higher diagnostic accuracy while trying to minimize all of the costs associated with traditional RGB-based methods [9].

Recent advances in deep learning and computer vision have enabled significant progress in automated medical imaging analysis, including dermatological applications. Semantic segmentation models like DeepLabv3+ have emerged as powerful tools for such tasks, leveraging encoder–decoder architectures and sophisticated feature extractors like Inception to capture global contextual and local spatial information. These capabilities make DeepLabv3+ particularly suitable for acne detection, enabling the precise delineation of lesions in complex visual data. We propose a hybrid model, DLI-Net, for acne detection by combining segmentation, classification, and explainable AI techniques. A diverse dataset of 12,338 images has been created to improve the model’s robustness and generalization.

- Firstly, an acne image dataset [10]. has been utilized, originally consisting of 1725 images of inflammatory acne, 594 images of non-inflammatory acne, and 8460 images of clear skin. To address the class imbalance, oversampling techniques such as data augmentation (rotation and flipping) were applied, resulting in a balanced dataset with 5051 images of inflammatory acne, 1962 images of non-inflammatory acne, and 5325 images of clear skin. This balanced dataset enhances model training, ensuring better classification reliability and generalization.
- Secondly, we integrate DeepLabV3 for segmentation and a modified InceptionV3 for classification. DeepLabV3 accurately segments acne-affected regions, and the classifier processes these regions to distinguish between inflammatory and non-inflammatory acne types, significantly improving detection accuracy.
- Thirdly, we conduct extensive experiments and comparative analyses with four baseline models to validate the superiority of DLI-Net. To optimize training efficiency, we utilize mixed-precision (FP16) computations, the AdamW optimizer, and a OneCycleLR scheduler, enabling faster convergence. Our evaluations demonstrate that DLI-Net effectively handles subtle inter-class similarities that challenge classification.
- Finally, we apply Grad-CAM to visualize the critical regions influencing the model’s predictions. This enhances interpretability and provides valuable insights into the decision-making process, fostering transparency and trust in the model’s outputs.

This paper is structured as follows: Section 2 provides a comprehensive review of relevant literature, summarizing the prior research in the field. Section 3 offers a detailed description of the proposed system model, outlining the methodology used to achieve the research objectives. In Section 4, we present an in-depth analysis of the experimental results, highlighting the effectiveness of the proposed approach. Finally, Section 5 concludes the paper, summarizing the key findings and contributions.

## 2. Literature Review

Deep learning (DL) has significantly transformed acne detection research, offering improvements in accuracy and automation. Nonetheless, persistent challenges remain concerning model interpretability, robustness across diverse populations, and the integration of fine-grained and contextual features. This section critically examines the existing literature in chronological order to position the contribution of the proposed DLI-Net framework.

A study by Rashataprucksa et al. [11] applied deep neural networks for acne detection using region-based object detection models. The approach showed promise, but its reliance on limited datasets hindered performance on diverse acne presentations, raising concerns about generalizability. In response, a study by Islam et al. [12] proposed a dual-integrated CNN model with contrast enhancement, achieving 97.53% high accuracy. The framework, however, lacked embedded interpretability mechanisms, which are essential for transparent clinical decision making. This shortfall highlights the need for frameworks that not only perform well but also provide explainable results for clinical adoption. Addressing the gap in interpretability, research by Femi et al. [13] introduced a web-based fuzzy logic system for severe acne diagnosis. Despite the advantage of rule-based interpretability, the system demonstrated limited scalability and was less effective for complex image-based classification tasks. Similarly, research by Quattrini et al. [14] proposed an interpretable CNN-based framework for acne severity assessment. Although the model met the demand for explainability, it suffered from computational inefficiencies that restricted real-time deployments, thus underscoring the challenge of balancing interpretability with real-time applicability. Further advancements emerged from the work by Junayed et al. [15], who developed ScarNet—a CNN tailored for acne scar classification that achieved 92.53% accuracy. The model emphasized dataset specificity but lacked cross-domain adaptability, which is a critical feature for clinical applications. In addition, inference speed and deployment feasibility were not adequately addressed, emphasizing the limitations of specialized models. Building on these ideas, research by Smith et al. [16] discussed AI-based advancements in acne detection and emphasized lightweight architectures. Their framework, however, remained conceptual with limited empirical validation, pointing to the need for more robust and validated solutions. In a similar vein, research by Lee et al. [17] presented an overview of DL-based acne detection methods, identifying the potential of CNNs while noted insufficient focus on explainable AI (XAI) integration in current models. Additional research by Kim et al. [18] examined transfer learning applications in dermatology. Although these methods offered improved model generalization, they often lacked fine-grained control over feature-level interpretation—an aspect crucial for dermatological diagnostics. In contrast, research by Wang et al. [19] proposed a hybrid CNN model for acne detection, which improved classification accuracy. Nonetheless, the model lacked explainability and contextual understanding, making it less suitable for clinical adoption. This gap in contextual understanding has been a recurring challenge in the literature, limiting the broader applicability of many models. Moreover, research by Zhang et al. [20] provided a survey of DL models for acne detection and highlighted performance-oriented optimizations.The absence of integration with patient metadata and interpretability frameworks, however, was a significant limitation. In an effort to enhance data diversity, research by Sun et al. [21] employed generative adversarial networks (GANs) to augment acne classification. Although the method improved data diversity, it introduced training instability and interpretability concerns, further complicating its real-world application.Regarding clinical integration, research by Wang et al. [22] implemented a machine learning-based acne grading system aligned with clinical standards. The model was effective in classification but lacked modular transparency and dynamic learning capabilities. Similarly, research by Li et al. [23] explored CNNs for acne scar detection and evaluation. High segmentation accuracy was achieved, yet real-time adaptability and reasoning capacity remained limited, reiterating the need for real-time decision-making features. Furthermore, research by Yao et al. [24] proposed a deep learning framework for acne classification with strong performance. Their approach primarily targeted static datasets without considering diverse real-world conditions, which limited its generalizability. Building on previous research, Chen et al. [25] introduced an enhanced CNN architecture with improved detection rates. The model, however, lacked multi-scale fusion and contextual reasoning capabilities, suggesting that a more holistic approach is necessary to capture the complexities of acne lesions. In addition, research by Wang et al. [26] examined image preprocessing techniques for acne detection, enhancing visual clarity and edge definition. Yet, the absence of downstream integration with classification models diminished clinical value, underscoring the need for an integrated solution. A comparative analysis by Li et al. [27] of acne classification models identified performance strengths but did not account for interpretability and fairness metrics. Finally, research by Wu et al. [28] proposed new methods for automated acne grading using advanced embeddings. Although the predictions were accurate in controlled environments, the model’s utility was limited in real-time, heterogeneous contexts, emphasizing the necessity for more robust and adaptable solutions in dynamic, real-world settings.

In summary, although the reviewed literature demonstrates significant progress in acne detection, notable gaps persist in three critical domains: (i) clinical interpretability and trust, (ii) robust generalization across populations and image conditions, and (iii) the integrated modeling of both local and global features. Furthermore, XAI remains underexplored across most frameworks.

This study proposes DLI-Net, a dual-stream hybrid CNN architecture that fuses lesion-specific and contextual features while embedding explainable modules, in order to address these limitations. By advancing both performance and transparency, DLI-Net aspires to bridge the gap between high-accuracy AI systems and their trustworthy application in real-world dermatological settings.

## 3. Methodology

### 3.1. Proposed Framework

**Figure 1:**
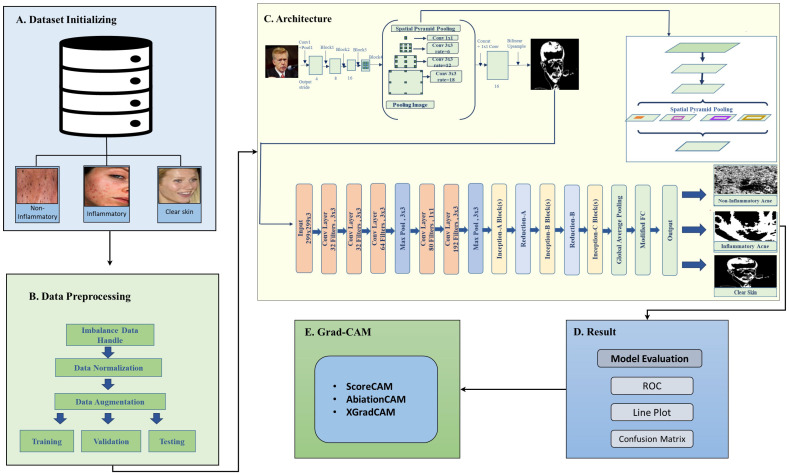
Proposed framework using acne imagery, image segmentation, image classification, and model evaluation.

The proposed deep learning framework for acne detection follows a structured four-stage approach to ensure accurate classification and clinical reliability, as illustrated in Figure 1. The process begins with dataset preparation (Figure 1A,B), utilizing an acne image dataset sourced from Kaggle, which includes three classes: inflammatory acne, non-inflammatory acne, and clear skin [10]. Due to class imbalance, where inflammatory and non-inflammatory acne have significantly fewer samples than clear skin, oversampling techniques are applied to enhance representation and prevent model bias. Once balanced, the dataset undergoes preprocessing steps such as normalization, noise reduction, and scaling to improve data quality and enhance feature extraction.

In the second stage (Figure 1C), a hybrid deep learning model is introduced, integrating DeepLabV3 for segmentation and InceptionV3 for classification. DeepLabV3 effectively isolates acne lesions by generating segmentation masks that highlight the affected regions, aiding in precise feature extraction. These segmentation outputs, illustrated in Figure 1, demonstrate the model’s capability to localize lesions accurately. The segmented regions are then classified using InceptionV3, which distinguishes between inflammatory and non-inflammatory acne, ensuring high classification accuracy.

The third stage (Figure 1D) involves a comprehensive evaluation of the model’s performance using key metrics such as accuracy, precision, recall, and computational efficiencies. These assessments validate the framework’s robustness and effectiveness in real-world clinical applications. Despite achieving high accuracy, deep learning models often lack interpretability, making it challenging to understand their decision-making process. To address this, the final stage (Figure 1E) incorporates Grad-CAM (Gradient-Weighted Class Activation Mapping) to enhance model transparency. Grad-CAM generates heatmaps that highlight the specific regions influencing the model’s predictions, ensuring that it focuses on acne-affected areas rather than unrelated regions. This approach improves trust and allows dermatologists to validate AI-generated results, making the framework more clinically applicable.

By integrating data balancing, advanced preprocessing, a hybrid deep learning model, and explainability techniques, the proposed framework provides a comprehensive and reliable solution for acne detection. Its high accuracy and interpretability contribute to improved diagnostic precision, making it valuable for both dermatology and telemedicine applications.

### 3.2. Image Acquisition

**Figure 2:**
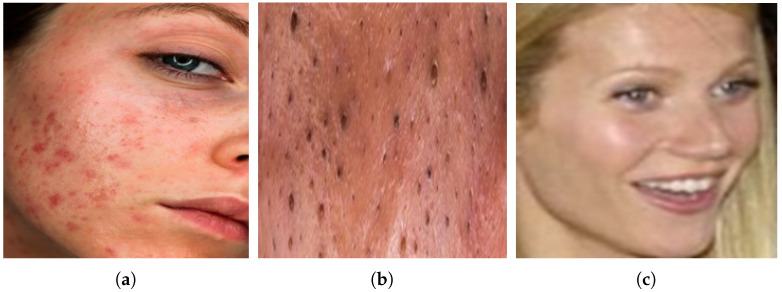
Sample images of acne types are shown. (**a**) Inflammatory acne. (**b**) Non-Inflammatory acne. (**c**) Clear skin.

The study utilizes an acne image dataset for the detection of two dermatological conditions: inflammatory acne and non-inflammatory acne, along with a third class representing clear skin. The dataset is sourced from Kaggle [10] and consists of 1725 images of inflammatory acne, 594 images of non-inflammatory acne, and 8460 images of clear skin. However, this dataset exhibits a significant class imbalance with a notably lower number of non-inflammatory and inflammatory acne images compared to the clear skin class. To address this imbalance and ensure the model learns effectively from all categories, oversampling techniques are applied. Specifically, data augmentation methods such as rotation and flipping are used to generate additional images, resulting in a newly constructed dataset with 5051 images of inflammatory acne, 1962 images of non-inflammatory acne, and 5325 images of clear skin. This balanced dataset is then utilized for model training, improving the model’s ability to distinguish between different acne types accurately. The distribution of images in the newly created dataset is presented in Table 1, while Figure 2 illustrates sample images from the dataset. The diverse nature of this dataset provides a strong foundation for deep learning-based dermatological models, ensuring better classification reliability and generalization, as supported by the findings of a self-constructed dataset contributed to improved performance in skin condition classification. By creating a more representative dataset, the study enhances the model’s ability to generalize across different skin types and populations, ultimately improving its clinical applicability and robustness in real-world dermatological assessments. Sample images of acne types are shown in Figure 2.

### 3.3. Data Preprocessing

A well-organized data preprocessing and augmentation pipeline was established to ensure effective model training and generalizability. The dataset was splitted into training (70%), validation (15%), and test (15%) subsets using stratified sampling to preserve the original distribution across the three classes: inflammatory acne, non-inflammatory acne, and clear skin. This ensured balanced representation and minimized sampling bias throughout the training and evaluation phases. All images were resized uniformly to 299×299 pixels to match the input requirements of the model architecture. Pixel intensities were normalized using ImageNet’s mean and standard deviation, facilitating stable learning dynamics. To enhance the diversity of the training dataset and improve model generalization, various augmentation techniques were applied. These included random horizontal and vertical flips (with a probability of 0.5), random rotations (up to ±30°) with a probability of 0.3, color jittering (brightness, contrast, saturation, and hue) with a probability of 0.2, and random erasing (with a probability of 0.1). These augmentations, applied only to the training set, introduced realistic variability in image orientation, illumination, and skin tone. The effectiveness of these techniques in improving model robustness is demonstrated in the Comparative Analysis and Ablation Study, where augmentations led to a notable improvement in classification accuracy.

Class imbalance was addressed by oversampling the non-inflammatory and inflammatory acne category to ensure a balanced representation across all classes during training. A class-weighted cross-entropy loss function was also used, assigning higher penalties to misclassified minority samples and prevented bias toward the majority classes.

Training efficiency was further improved by enabling prefetching and optimizing data loading to reduce I/O latency. A batch size of 16 was adopted to balance memory utilization and gradient stability. Collectively, this preprocessing and augmentation strategy contributed to enhanced model robustness, improved class sensitivity, and reliable acne classification performance.

### 3.4. Proposed Models

Acne detection is performed using DLI-Net, which is a hybrid deep learning model combining DeepLabV3 for lesion segmentation and InceptionV3 for classification. This section introduces the proposed architecture for detecting acne and its types.

#### 3.4.1. Deep Learning Models

Deep learning models such as InceptRes101V2 [29], VGG-19 [30], DeepLabv3 [31], InceptionV3 [32], MobileNetV3 [33], ResNet50 [34], Vision Transformer (ViT) [35], and a proposed DLI-Net architecture are utilized for acne detection due to their advanced capabilities in feature extraction, segmentation, and classification. These models significantly enhance the accuracy and efficiency of identifying and analyzing acne types in images, including inflammatory and non-inflammatory categories. Leveraging these state-of-the-art architectures enables scalable and automated diagnostic processes, ensuring reliable performance across diverse datasets and environmental conditions. The integration of multiple architectures provides a robust framework for comprehensive acne detection and analysis.

#### 3.4.2. DeepLabV3 Model Architecture for Acne Detection

**Figure 3:**
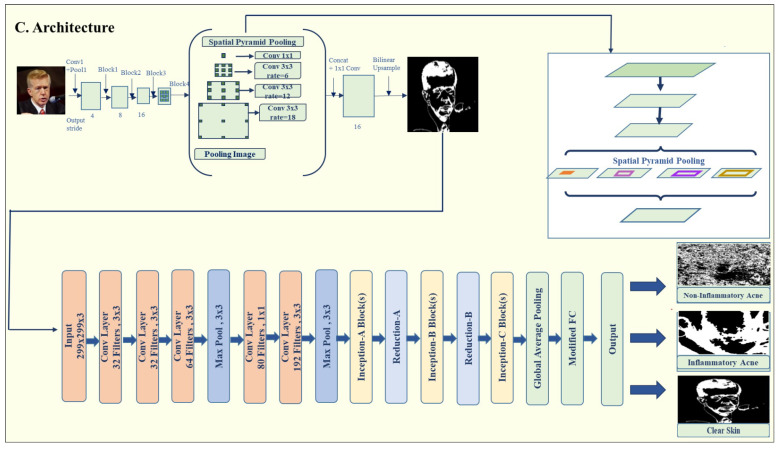
Proposed model architecture.

The DeepLabV3 architecture shown in Figure 3, introduced by Chen et al. [25], is a state-of-the-art model for semantic image segmentation and is particularly effective for detecting acne lesions. The architecture is composed of several key stages: Encoder (Feature Extraction), Atrous Spatial Pyramid Pooling (ASPP), Combining Encoder and ASPP, Decoder (Up-sampling), and Output Layer. Below is a breakdown of each stage shown in Table 2.

Stage 1: Encoder (Feature Extraction). The encoder’s primary role is to extract features from the input images. This is achieved through the pre-trained ResNet50 backbone in the DeepLabV3 model from the torch.vision.models. Segmentation module. The encoder progressively reduces the spatial resolution of the input while capturing critical low-level and high-level features. These features are essential for differentiating acne lesions from healthy skin. The input images are first resized to (299, 299) and undergo extensive augmentation. As the encoder processes the images through its convolutional layers, the spatial resolution of the feature maps decreases while essential features, both low-level and high-level, are preserved. This step ensures that critical information, such as lesion boundaries and texture, is retained. The encoder progressively reduces spatial dimensions, starting from 128 × 128 and reaching a resolution by the time it passes through the ASPP module.

Stage 2: Atrous Spatial Pyramid Pooling (ASPP). The ASPP module captures a multi-scale context by using atrous convolutions (also known as dilated convolutions) with various dilation rates. This allows the model to capture features from different receptive fields without compromising spatial resolution. ASPP is handled by the convolutional layers in the DeepLabV3 implementation, which refine the feature map, allowing the model to understand acne lesions at different scales. This is key to distinguishing various acne types based on their size and location.

Stage 3: Combining Encoder and ASPP. The high-resolution feature maps obtained from the ASPP module are merged with features extracted by the encoder. This fusion ensures that the model benefits from both fine-grained spatial details and broad contextual information. The combined feature maps are subsequently used for generating a segmentation map, which outlines the acne lesions on the input images.

Stage 4: Decoder (Up-sampling). The decoder reconstructs the spatial resolution of the feature maps to match the input image dimensions. The segmentation map produced by the DeepLabV3 model is concatenated with the input image to create a combined feature map. A channel reducer, implemented as a 1 × 1 convolution, adjusts the number of channels in the combined feature map before passing it to the classification module. This step ensures that the segmentation and classification components of the model are seamlessly integrated. The decoder progressively restores the spatial resolution through up-sampling, refining the feature maps with each step. For example, resolutions are increased from 4 × 4 to 128 × 128 through successive up-sampling layers in Table 3, preserving the important details necessary for accurate segmentation. The final segmentation map is generated using a 1 × 1 convolution and a sigmoid activation function, producing a binary mask to identify acne lesions. To facilitate seamless integration, the final segmentation map generated by DeepLabV3 is concatenated with the original input image, and this fused feature map is passed through a 1 × 1 convolution (channel reducer) before being forwarded to the InceptionV3 classification head, enabling the early fusion of spatial lesion features with the raw input.

#### 3.4.3. Modified-Inception V3 Model

For classification, we utilize a modified InceptionV3 model pre-trained on ImageNet by Szegedy et al. [29], to enhance acne detection capabilities. Firstly, we removed the auxiliary classifiers (aux_logits = False) that are typically included in the original InceptionV3 architecture. This change aimed to reduce the computational cost and focus solely on the primary classification output, as auxiliary classifiers are primarily beneficial for multi-class tasks but were not necessary for this binary classification task. Secondly, we customized the fully connected (FC) layer of the model to better suit the acne detection problem. We replaced the original FC layer with a sequential block consisting of a linear layer with 512 units, which was followed by a ReLU activation function and a dropout layer (set to 0.4) to reduce overfitting. This was concluded by another linear layer, which outputs the final classification score for each of the acne categories. Thirdly, the classification module utilizes a modified InceptionV3 model, which receives the fused input composed of the original image concatenated with the lesion segmentation output from DeepLabV3. This early fusion approach enhances classification accuracy by enabling the model to leverage lesion-focused spatial features from the segmentation stage. This demonstrates that the early fusion of lesion-aware features from DeepLabV3 with the raw input significantly boosts the classification performance of the InceptionV3 head, emphasizing the effectiveness of functional integration in the proposed hybrid architecture. Finally, we utilized pre-trained weights from a large and diverse dataset. This significantly reduced the training time and improved the model’s ability to generalize acne detection, as the pre-trained model had already learned meaningful features from millions of images.

- Fully Connected Layer: The fully connected (FC) layer in a neural network maps the input features to the final output space. In this case, the modified InceptionV3 model uses two linear layers, where the first layer has 512 units, which is followed by a ReLU activation and a dropout layer to reduce overfitting. The output of the first linear layer is passed through ReLU and then through the second linear layer to produce the classification results. This structure helps to learn complex relationships in the data by transforming the high-dimensional feature map into a desired output size.

The mathematical representation for the FC layer is

(1) $$y=W_{2}\cdot \mathrm{ReLU}\left(W_{1}\cdot x+b_{1}\right)+b_{2}$$

**Figure 4:**
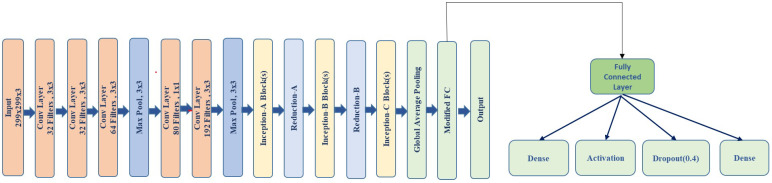
InceptionV3 modified layer.

This hybrid model DLI-Net integrates segmentation using DeepLabV3 and classification with the modified InceptionV3 (shown in Figure 4) to provide the accurate detection and categorization of acne lesions. The segmentation map enhances classification by providing spatial cues, improving the model’s robustness for clinical applications.

### 3.5. Training Objectives and Optimization Strategies

The optimization of the DLI-Net architecture was achieved through task-specific objective functions and training strategies. The classification branch utilized categorical cross-entropy loss, which is a widely used approach for multi-class classification tasks. This loss function measures the dissimilarity between the predicted probabilities and the ground truth class labels.

(2) $$L\left(y,\hat{y}\right)=-\frac{1}{N}\sum_{i=1}^{N}\sum_{c=1}^{C}y_{i,c}\log\left({\hat{y}}_{i,c}\right)$$

To update model weights, gradient descent was employed. Mixed precision training using auto-cast and GradScaler was integrated to enhance computational efficiency while preserving accuracy. The standard weight update rule is defined as

(3) ω=ω−η∇L(ω)

Learning rate adaptation was achieved using the OneCycleLR scheduling strategy, which enables a cyclic learning rate trajectory to facilitate faster convergence and robust generalization. The scheduler’s update mechanism is defined as

(4) $$\eta \left(t\right)=\eta_{\min}+\frac{1}{2}\left(\eta_{\max}-\eta_{\min}\right)\left(1+\cos\left(\frac{\pi t}{T}\right)\right)$$

For the segmentation branch, the binary cross-entropy loss was applied, which is suitable for learning pixel-wise binary masks corresponding to acne-affected regions. It is formulated as

(5) $$L_{\mathrm{BCE}}\left(y,\hat{y}\right)=-\frac{1}{N}\sum_{i=1}^{N}\left(y_{i}\log\left({\hat{y}}_{i}\right)+\left(1-y_{i}\right)\log\left(1-{\hat{y}}_{i}\right)\right)$$

### 3.6. Training Hyperparameters and Setup

The DLI-Net framework was trained with a carefully selected configuration of hyperparameters to achieve stable convergence and optimal performance. AdamW was chosen as the optimizer for its enhanced weight decay regularization compared to standard Adam optimizers. The learning rate was dynamically controlled using the OneCycleLR scheduler with both the initial and maximum values set to $1\times 10^{-4}$.

A batch size of 16 was employed to maintain gradient stability and compatibility with hardware constraints. The training was carried out over 30 epochs with continual performance monitoring on a stratified validation set. The classification and segmentation branches were jointly optimized using categorical cross-entropy and binary cross-entropy loss functions, respectively.

A comprehensive summary of the training hyperparameters used in this study is provided in Table 4.

## 4. Evaluation Metrics

In evaluating the performance of an acne detection model, particularly for distinguishing inflammatory and non-inflammatory acne and segmenting affected regions, the choice of appropriate metrics is crucial. For classification tasks, Precision (6), Recall (7), and the F1 score (8), which balances precision and recall, are key metrics [36]. Precision ensures that the model effectively identifies acne-affected regions while minimizing false positives. Recall measures the model’s ability to correctly detect true acne cases, ensuring that diverse acne types are identified. The F1 score provides an overall evaluation by balancing precision and recall.

(6) $$Precision=\frac{TP}{TP+FP}$$

where TP=TruePositive,FP=FalsePositive.

(7) $$Recall=\frac{TP}{TP+FN}$$

where TP=TruePositive,FN=FalseNegative.

(8) $$F1\;Score=2\times \frac{Precision\times Recall}{Precision+Recall}$$

(9) $$IoU=\frac{A_{o}}{A_{u}}$$

where IoU = Intersection over Union, $A_{O}$ = Area of Acne Overlap, and $A_{u}$ = Area of Acne. For segmentation,

(10) $$D=\frac{2|X\cap Y|}{\left|X|+|Y\right|}$$

where *D* is the Dice Coefficient, |X∩Y| is the intersection, and |X| and |Y| are the sizes of pixel sets. The Intersection over Union (IoU) (9) and the Dice Coefficient (10) are used to assess the accuracy of predicting acne-affected areas. IoU measures the overlap between the predicted and actual acne-affected regions, ensuring a precise delineation of boundaries. Meanwhile, the Dice Coefficient quantifies the similarity between the predicted and ground-truth segmentation with a Dvalue of 1 indicating perfect alignment and 0 denoting no overlap.

### 4.1. Proposed Model Outcome

**Figure 5:**
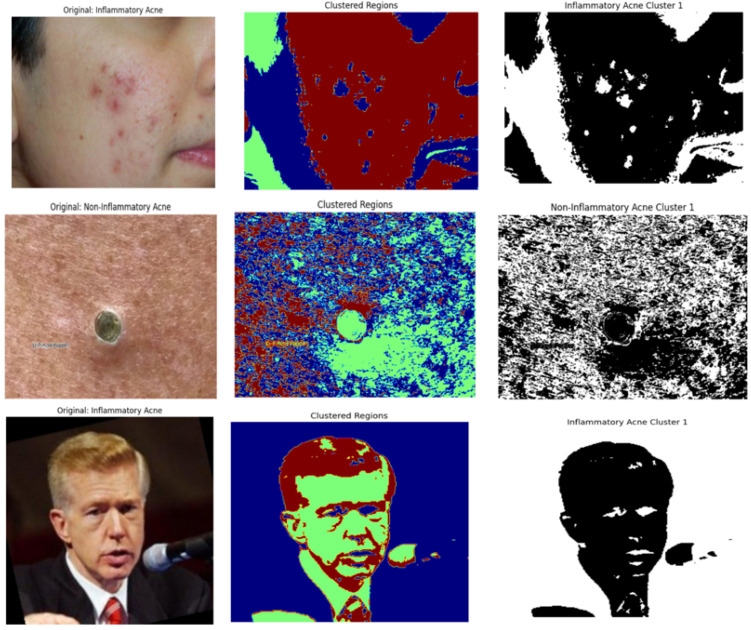
Segmentation masks for three distinct categories: inflammatory, non-inflammatory and clear skin.

This section presents the results of segmentation and classification models developed for acne detection shown in Figure 5. The performance of these models has been evaluated using the metrics and the formulas detailed.

**Figure 6:**
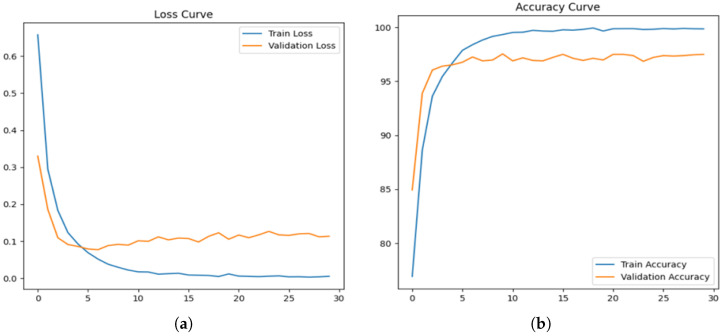
Loss curve and accuracy curve for proposed DLI-Net model. (**a**) Loss Curve of Proposed DLI-Net model. (**b**) Accuracy Curve of Proposed DLI-Net model.

Figure 6a illustrates the loss curve for the acne detection model, showing a sharp decline in training loss during the initial epochs, signifying effective learning of the data’s patterns. The validation loss, while slightly higher than the training loss, stabilizes over time, indicating that the model achieves a good balance without significant overfitting. Meanwhile, Figure 6b presents the accuracy curve, where the training accuracy approaches near perfection as the epochs progress. The validation accuracy steadily increases, stabilizing between 96 and 98%, reflecting the model’s strong generalization ability in correctly identifying acne-related features across unseen data.

**Figure 7:**
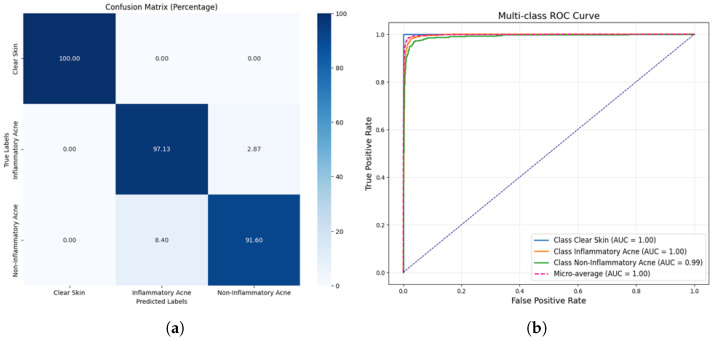
Confusion matrix and ROC curve for proposed DLI-Net model. (**a**) Confusion matrix proposed DLI-Net model. (**b**) ROC of Proposed DLI-Net model.

Figure 7a presents the confusion matrix for the acne detection model, highlighting its performance in classifying clear skin, inflammatory acne, and non-inflammatory acne. The model achieves a flawless classification of clear skin with 100% accuracy, reflecting its robustness in identifying non-acne cases. For inflammatory acne, the model demonstrates an excellent prediction rate of 97.13% with a minimal misclassification rate of 2.87% in the non-inflammatory acne category. Similarly, non-inflammatory acne is predicted with 91.60% accuracy, although 8.40% of instances are misclassified as inflammatory acne. These results underline the model’s strong overall performance, particularly its ability to distinguish clear skin, while minor overlaps in acne classifications indicate areas for further refinements. Figure 7b shows the multi-class ROC curve for classifying clear Skin, inflammatory acne, and non-inflammatory acne, with AUCs of 1.00, 1.00, and 0.99, respectively. The model demonstrates near-perfect performance, with the micro-average AUC also at 1.00, highlighting its exceptional classification accuracy.

### 4.2. Model Testing Performance

Table 5 presents a comparative evaluation of various deep learning architectures used for acne detection. The performance metrics include weighted average F1 score, precision, recall, and validation accuracy. The proposed hybrid DLI-Net model demonstrates superior performance across all evaluated criteria by synergistically integrating segmentation and classification within a unified framework.

As observed in Table 5, the proposed hybrid DLI-Net model consistently outperforms traditional classifiers and segmentation networks. Its architecture combines DeepLabV3-based spatial segmentation with a fine-tuned InceptionV3 classifier, facilitating both lesion localization and acne type classification. The model achieves a high validation accuracy of 97% with balanced precision and recall, confirming its reliability across diverse input conditions.

Compared to standalone classifiers such as InceptionV3, ResNet50, and MobileNetV3, which lack spatial contextual awareness, DLI-Net’s integrated design enables enhanced interpretability and lesion-specific decision making. Although Vision Transformers (ViTs) offer promising performance, their absence of spatial segmentation components limits their clinical applicability. Similarly, DeepLabV3 achieves strong recall due to its segmentation strength but lacks dedicated classification capabilities. Legacy architectures such as VGG-19 underperform due to limited feature representation capacity.

### 4.3. Class-Wise Performance Evaluation and Handling Imbalanced Data

Class-wise metrics were analyzed in terms of precision, recall, and F1 score to evaluate the model’s performance across all acne categories. The results, summarized in Table 6, demonstrate the model’s ability to distinguish subtle patterns in imbalanced datasets.

The model achieves perfect performance on the clear skin class, indicating robust specificity in detecting acne-free regions. Inflammatory acne, typically characterized by more prominent visual cues, is classified with a high F1 score of 0.97. Notably, the model achieves a strong F1 score of 0.92 for non-inflammatory acne, which is a class historically prone to misclassification due to its subtle visual characteristics.

This improvement can be attributed to a combination of targeted oversampling strategies and the application of a class-weighted loss function. Prior to oversampling, the F1 score for non-inflammatory acne was observed to be approximately 0.85. The integration of these data-level and algorithm-level interventions significantly enhanced sensitivity and balanced learning across all classes.

The results demonstrate the model’s robustness against class imbalance and reinforce its suitability for deployment in real-world clinical settings where category distribution is rarely uniform. Moreover, consistent performance across all classes ensures fair representation and diagnostic reliability, which are critical in medical decision support systems.

### 4.4. Ablation Study and Component-Wise Evaluation

An extensive ablation study was conducted to further validate the design choices of the proposed DLI-Net framework by systematically removing or replacing individual components. The evaluation included model variants without segmentation integration and substitutions of the modified InceptionV3 classifier with alternative architectures such as DenseNet [37], ResNet, ViT, and EfficientNetB0 [38].

Table 7 presents the comparative performance of these variants based on accuracy, precision, recall, and F1 score. The analysis clearly illustrates that the combination of DeepLabV3 segmentation with the modified InceptionV3 classifier yields the highest performance, validating the efficacy of our hybrid architecture.

In the absence of the segmentation branch (using classification-only variants), a consistent drop in accuracy and class-wise F1 score was observed. Specifically, using only the modified InceptionV3 classifier resulted in a reduced accuracy of 95.41% and a lower F1 score of 0.92 for the non-inflammatory acne class, highlighting the contribution of segmentation features in enhancing acne region localization and classification sensitivity.

Similarly, replacing the classification module with other backbones such as ViT, DenseNet, or EfficientNetB0 led to moderate declines in performance. The best-performing alternatives among these were DeepLabV3 combined with EfficientNetB0 and DenseNet, achieving accuracies of 96.65% and 96.60%, respectively. However, none surpassed the 97.30% accuracy and overall F1 score of 0.973 achieved by the full DLI-Net configuration.

These results affirm that both a segmentation-driven feature enhancement and the architectural optimization of the classification head are critical for achieving robust and balanced acne detection performance. This demonstrates that the early fusion of lesion-aware features from DeepLabV3 with the raw input significantly boosts the classification performance of the InceptionV3 head, emphasizing the effectiveness of functional integration in the proposed hybrid architecture.

### 4.5. Explainable AI (XAI) Using Class Activation Mapping Techniques

**Figure 8:**
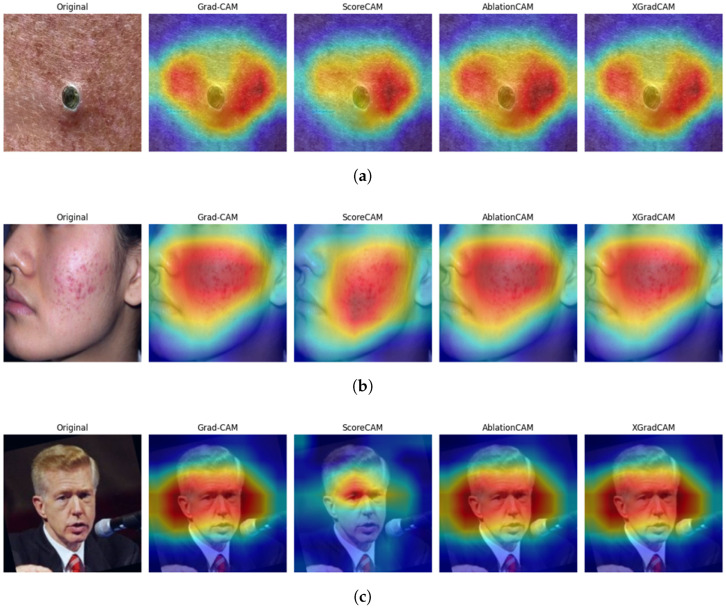
XAI heatmaps for non-inflammatory acne, inflammatory acne, and clear skin using Grad-CAM, ScoreCAM, AblationCAM, and XGradCAM. (**a**) Non-Inflammatory. (**b**) Inflammatory. (**c**) Clear Skin.

Explainable AI (XAI) methods were integrated into the proposed model to enhance its interpretability and clinical reliability. Specifically, Gradient-Weighted Class Activation Mapping (Grad-CAM) [39], ScoreCAM [40], AblationCAM [41], and XGradCAM [42] were employed. These techniques visualize the model’s internal reasoning by highlighting spatial regions within the input image that most strongly influence its classification decisions shown in Figure 8.

Grad-CAM generates a heatmap by computing the gradients of the target class score with respect to the feature maps of the last convolutional layer. The importance weights $\alpha_{k}^{c}$ for each feature map $A^{k}$ are calculated as

(11) $$\alpha_{k}^{c}=\frac{1}{Z}\sum_{i}\sum_{j}\frac{\partial y^{c}}{\partial A_{ij}^{k}}$$

where *Z* denotes the number of pixels in the feature map, $y^{c}$ is the score for class *c*, and $A_{ij}^{k}$ represents the activation at position (i,j) in the *k*-th feature map. The final Grad-CAM output heatmap $L_{\mathrm{Grad}-\mathrm{CAM}}^{c}$ is computed as

(12) $$L_{\mathrm{Grad}-\mathrm{CAM}}^{c}=\mathrm{ReLU}\left(\sum_{k}\alpha_{k}^{c}A^{k}\right)$$

ScoreCAM is a gradient-free method that assesses activation maps by perturbing the input image and analyzing confidence scores, producing smoother and more robust heatmaps. AblationCAM isolates critical activations by selectively ablating feature maps, while XGradCAM enhances spatial focus by applying an element-wise product of gradients and activation maps, yielding more compact and clinically relevant heatmaps.

Applied to test images across all acne severity levels, Grad-CAM highlighted lesion cores, ScoreCAM captured broader lesion peripheries, AblationCAM identified key disease indicators, and XGradCAM provided precise boundary activations. In class-wise evaluation, Grad-CAM emphasized comedones and inflamed clusters, while ScoreCAM and XGradCAM effectively captured lesion edges and skin texture variations. For Clear Skin images, all methods exhibited low or random activation, reinforcing the model’s discrimination ability.

These visual explanations confirm that predictions are based on medically relevant features, enhancing interpretability and clinical trust. Future work may explore quantitative validation using pixel-level lesion annotations or dermatological segmentation masks to further refine explainability.

### 4.6. Comparative Analysis

The results of our proposed Hybrid Model DLI-Net for acne detection and segmentation provide a comprehensive approach by combining classification, image segmentation, and augmentation techniques. This model outperforms existing studies in several key areas shows in Table 8. For example, Islam et al. [12] integrated classification and segmentation but did not include the hybrid model or data augmentation, which limits its robustness and performance in handling diverse acne conditions. Similarly, Chen et al. [25] introduced an enhanced CNN framework for acne detection but did not incorporate multi-scale feature fusion, which may have limited the model’s effectiveness in capturing lesions of varying sizes and complexities. Our Hybrid Model DLI-Net addresses these gaps by incorporating both the segmentation and classification components along with a robust augmentation strategy, achieving superior performance in classification and segmentation, as demonstrated by its high accuracy and F1 score.

In comparison, Rashataprucksa et al. [11] proposed a model that combines classification and segmentation but did not include a hybrid approach or data augmentation, which limits the model’s ability to handle complex acne patterns effectively Furthermore, Femi et al. [13] focused solely on classification without segmentation or augmentation, which reduces their ability to both identify and accurately locate acne lesions in real-world scenarios. The lack of segmentation and augmentation in their models also means they may not generalize as well to diverse and unseen data. Our Hybrid Model DLI-Net, by combining all these essential elements, addresses the limitations found in these prior works, offering an advanced solution with enhanced accuracy and reliability across various acne types.

In contrast to Islam et al. [12], Chen et al. [25], and Rashataprucksa et al. [11], whose models focus on one or two components, our Hybrid Model DLI-Net stands out for its comprehensive integration of classification, segmentation, and augmentation These elements work together to improve the model’s generalization and robustness. The inclusion of augmentation further strengthens the model’s ability to avoid over-fitting, which is a common challenge faced by models lacking this technique. As highlighted by Wu et al. [28], data augmentation is critical for ensuring that the model performs well on unseen data. Thus, the Hybrid Model DLI-Net demonstrates a more complete and effective solution for acne classification and segmentation, combining the best aspects of previous research while addressing their limitations.

### 4.7. Comparison of FP16 vs. FP32 Precision Modes

The computational efficiency and inference stability of the proposed DLI-Net model were evaluated by training it under two different precision configurations: standard full-precision (FP32) and mixed-precision (FP16). This comparison examines both the model’s predictive performance and training efficiency, offering insights into optimal deployment strategies in resource-constrained environments.

Table 9 presents a detailed comparison of test accuracy, precision, recall, F1 score, and total training time across the two precision settings. The FP32-based model achieved a marginally superior test accuracy of 97.30% and F1 score of 0.973 compared to 96.49% and 0.965, respectively, in the FP16 configuration. The class-wise analysis further showed that the FP32 model exhibited improved sensitivity, particularly for the non-inflammatory acne category, where the F1 score increased from 0.90 to 0.92.

Although FP16 training benefits from reduced memory usage and potentially faster computation through tensor core acceleration, the training time remained relatively similar to FP32 due to initialization overhead and GPU scheduling latency. However, minor fluctuations in class-wise recall and precision suggest that mixed-precision training may slightly affect gradient precision and convergence stability, especially in fine-grained multi-class classification tasks like acne detection.

These findings indicate that while FP16 is suitable for rapid prototyping and deployment in edge devices, the FP32 configuration offers superior reliability and slightly improved classification consistency. Given these observations, FP32 was selected as the final deployment configuration for the DLI-Net framework, ensuring optimal trade-offs between computational cost and clinical-grade accuracy.

### 4.8. Clinical Applicability and Limitations

DLI-Net demonstrates strong potential as a diagnostic aid for dermatologists, integrating lesion segmentation and classification to provide a comprehensive analysis of acne characteristics such as morphology, distribution, and severity. These capabilities enable rapid screening, clinical decision support, and disease monitoring, particularly in resource-limited or teledermatology applications.

The model’s explainability through Grad-CAM, ScoreCAM, AblationCAM, and XGradCAM enhances transparency, ensuring that predictions are based on medically relevant features rather than arbitrary patterns. This fosters trust in AI-assisted diagnosis and supports its integration into clinical workflows.

However, certain challenges must be addressed for real-world deployment. Dataset diversity remains a limitation, as the current dataset lacks a wide representation of different skin tones, age groups, and ethnic backgrounds, potentially affecting generalizability. Additionally, variations in lighting, image resolution, and real-world artifacts could impact model robustness. The computational cost of DLI-Net is also high, limiting its usability in mobile or low-resource environments without further optimization.

DLI-Net is designed to augment, not replace, clinical expertise by providing consistent, explainable decision support. With further validation, regulatory assessment, and model optimizations, it could be seamlessly integrated into dermatology practices to improve diagnostic accuracy and reduce inter-observer variability.

## 5. Conclusions

This study introduced DLI-Net, a hybrid deep learning framework combining DeepLabV3 for lesion segmentation and a Modified InceptionV3 for acne classification. The model demonstrated high accuracy (97%) and a strong F1 score (0.973), outperforming alternative architectures in acne detection. By incorporating explainability techniques, we ensured that model predictions aligned with clinically relevant features, enhancing transparency and usability in dermatological diagnosis.

Despite its strong performance, certain limitations remain. Dataset diversity constraints could affect generalizability, and image variations in real-world settings may introduce challenges in deployment. While oversampling and class-weighted loss mitigated class imbalance, further refinements are needed to prevent potential overfitting. Moreover, DLI-Net’s computational complexity restricts its scalability for real-time applications, necessitating model compression and lightweight adaptations.

Future work will focus on enhancing generalization and scalability. Expanding the dataset with diverse acne types and imaging conditions will strengthen robustness across populations. To reduce computational overhead, we will explore model pruning, quantization, and lightweight architectures for edge and mobile deployment. Additionally, advanced explainability methods and quantitative validation using expert-annotated lesion maps will be investigated to further enhance interpretability and clinical trust. These improvements will pave the way for real-world adoption and potential integration into dermatology workflows to support accurate, AI-assisted acne diagnosis.

## Figures and Tables

**Table 1:** Dataset details of acne types.

Classes	Total Images
Inflammatory	5051
Non-Inflammatory	1962
Clear Skin	5325
Total	12,338

**Table 2:** Encoder feature layers in the DeeplabV3 model.

Layer Name	Dimensions
Initial Conv Block	128 × 128
Inception Block 1	64 × 64
Inception Block 2	32 × 32
Inception Block 3	16 × 16
Atrous Block 1 (DeepLabV3)	8 × 8
Atrous Block 2 (DeepLabV3)	4 × 4
ASPP Module	4 × 4

**Table 3:** Up-sampling blocks in the DeeplabV3 decoder.

Up-Sampling Stage	Dimensions	Number of Filters
Stage 1	4 × 4 to 8 × 8	512
Stage 2	8 × 8 to 16 × 16	256
Stage 3	16 × 16 to 32 × 32	128
Stage 4	32 × 32 to 64 × 64	64
Stage 5	64 × 64 to 128 × 128	32

**Table 4:** Training configuration and hyperparameters used for DLI-Net framework.

Hyperparameter	Value
Optimizer	AdamW
Learning Rate	Initial = $1\times 10^{-4}$, Maximum (OneCycleLR) = $1\times 10^{-4}$
Learning Rate Scheduler	OneCycleLR
Batch Size	16
Number of Epochs	30
Classification Loss Function	Cross-Entropy Loss
Segmentation Loss Function	Binary Cross-Entropy Loss
Data Augmentation	Horizontal Flip, Vertical Flip, Rotation (±30°), Color Jitter, Random Erasing
Normalization	Mean = [0.485, 0.456, 0.406]; Std = [0.229, 0.224, 0.225] (ImageNet-based)
Image Resolution	299 × 299 pixels
Training–Validation–Test Split	70%–15%–15% (stratified sampling)

**Table 5:** Model-wise performance comparison based on weighted average metrics.

Model	F1 Score	Precision	Recall	Validation Accuracy
Hybrid DLI-Net (Proposed)	0.97	0.97	0.97	0.97
InceptionV3	0.94	0.94	0.94	0.94
MobileNetV3	0.93	0.94	0.93	0.93
ResNet50	0.94	0.94	0.94	0.94
ViT	0.92	0.92	0.92	0.92
DeepLabV3	0.94	0.94	0.95	0.95
VGG-19	0.65	0.78	0.67	0.83
InceptionResNet101V2	0.93	0.95	0.91	0.92

**Table 6:** Class-wise precision, recall, and F1 score for DLI-Net on test data.

Class	Precision	Recall	F1-Score
Clear Skin	1.00	1.00	1.00
Inflammatory Acne	0.98	0.95	0.97
Non-Inflammatory Acne	0.89	0.95	0.92

**Table 7:** Ablation study results comparing different architectural variants of DLI-Net.

Model Configuration	Accuracy	Precision	Recall	F1-Score
DLI-Net (Proposed Model)	97.30%	0.974	0.973	0.973
Modified InceptionV3	95.41%	0.94	0.95	0.95
DeepLabV3 Only	95.00%	0.94	0.95	0.94
DeepLabV3 + Pre-Trained InceptionV3	96.65%	0.967	0.966	0.967
Pre-Trained InceptionV3	94.29%	0.87	0.88	0.87
DeepLabV3 + ViT	95.00%	0.95	0.95	0.95
DeepLabV3 + DenseNet	96.60%	0.966	0.966	0.966
DeepLabV3 + EfficientNetB0	96.43%	0.963	0.964	0.963

**Table 8:** Comparative analysis for acne detection.

Authors	Classification	Image Segment	Hybrid Model DLI-Net	Aug	Model Compare
Proposed DeeplabV3 Model	✓	✓	✓	✓	✓
Islam et al. (2022) [12]	✓	X	X	✓	X
Rashataprucksa et al. (2020) [11]	✓	X	X	X	X
Femi et al. (2020) [13]	✓	X	X	X	X

**Table 9:** Performance comparison between FP16 and FP32 precision modes.

Metric	FP16 (Mixed Precision)	FP32 (Standard Precision)
Test Accuracy	96.49%	97.30%
Precision (Weighted Avg)	0.967	0.974
Recall (Weighted Avg)	0.965	0.973
F1 Score (Weighted Avg)	0.965	0.973
Training Time (minutes)	329.75	327.30
F1 Score: Clear Skin	1.00	1.00
F1 Score: Inflammatory Acne	0.96	0.97
F1 Score: Non-Inflammatory Acne	0.90	0.92

## Data Availability

The data presented in this study are available on request from the corresponding author due to future publication purposes.
